# Challenges in enriching milk fat with polyunsaturated fatty acids

**DOI:** 10.1186/s40104-015-0025-0

**Published:** 2015-06-12

**Authors:** Jennifer Stamey Lanier, Benjamin A. Corl

**Affiliations:** Phibro Animal Health Corporation, Teaneck, NJ USA; Department of Dairy Science, Virginia Tech, Blacksburg, VA 24061-0315 USA

**Keywords:** Dairy cow, Milk fat, Polyunsaturated fatty acids

## Abstract

Milk fatty acid composition is determined by several factors including diet. The milk fatty acid profile of dairy cows is low in polyunsaturated fatty acids, especially those of the n-3 series. Efforts to change and influence fatty acid profile with longer chain polyunsaturated fatty acids have proven challenging. Several barriers prevent easy transfer of dietary polyunsaturated fatty acids to milk fat including rumen biohydrogenation and fatty acid esterification. The potential for cellular uptake and differences in fatty acid incorporation into milk fat might also have an effect, though this has received less research effort. Given physiological impediments to enriching milk fat with polyunsaturated fatty acids, manipulating the genome of the cow might provide a greater increase than diet alone, but this too may be challenged by the physiology of the cow.

## Introduction

Concentrations of milk components determine the value of milk produced. Non-nutritional factors like parity, season, genetics, stage of lactation, and mastitis are known to influence milk composition. Milk fat is the most variable and energy dense component of milk and is easily influenced by nutrition, followed by protein and lactose, respectively, which remain relatively stable [1]. Plant and marine oils, feeding strategy, dietary fiber, concentrate inclusion, and ionophores are known to alter milk fat content and composition. Even though intense genetic selection for milk yield decreases milk fat content, there is minimal effect of selection for milk yield on milk fatty acid profile [2, 3].

The ability to change milk composition allows for improved processing characteristics, enhanced nutrient composition, and delivery of nutraceuticals for human health. Increasing the level of polyunsaturated fatty acids is the goal of nutritional modulation of milk fat synthesis, whether for a physical characteristic, such as improving the spreadability of butter, or a nutritional benefit, like decreased milk fat content or enhanced polyunsaturated fatty acids concentration [1, 4]. Human health benefits associated with consuming n-3 fatty acids encourage development of functional foods for humans. Consumer perceptions of milk fat composition and quality may be improved by dietary supplements that increase milk fat content of n-3 fatty acids [4]; however, markedly increasing milk fat polyunsaturated fatty acid content has proved difficult.

### Ruminal biohydrogenation

Based on the diet of the cow, milk should be rich in polyunsaturated fatty acids. The diet of a lactating dairy cow typically contains 4–6 % fat and is enriched in polyunsaturated fatty acids [5]. The glycolipids and phospholipids found in forages are rich in linolenic (*cis*-9, *cis*-12, *cis*-15 18:3) and linoleic acids (*cis*-9, *cis*-12 18:2). In contrast, the lipids of seed oils found in concentrates, such as corn or soybeans, are predominantly triglycerides containing linoleic acid and oleic acid (*cis*-9 18:1); however, efforts in plant breeding have been made to change the fatty acid profile of seed oils to alter the relative proportions of saturated, monounsaturated, and polyunsaturated fatty acids [6, 7]. Although lipids are found in small amounts in ruminant feedstuffs, the fatty acids are largely unsaturated.

Despite significant amounts of polyunsaturated fatty acids entering the rumen, outflows of polyunsaturated fatty acids into the small intestine are limited. The major fatty acid entering the small intestine is stearic acid. Smaller quantities of unsaturated fatty acids, many containing *trans* double bonds are also found entering the small intestine [8]. This change in fatty acid profile from consumption to absorption has been repeatedly observed [9–11]. Significant quantities of dietary polyunsaturated fatty acids were converted to more saturated fatty acids prior to the small intestine due to rumen biohydrogenation.

Ester linkages in feed lipids are hydrolyzed by microbial lipases, and lipolysis is a prerequisite for biohydrogenation of dietary unsaturated fatty acids [12]. Bacteria that biohydrogenate dietary lipids act on free fatty acids, not esterified fatty acids [13, 14]. Several bacterial species are known to hydrolyze fatty acid ester linkages, and a full complement of lipases capable of hydrolyzing dietary lipids are found among rumen bacteria [15–17].

A range of diverse rumen bacteria have been isolated that have the capacity to biohydrogenate unsaturated fatty acids with the protozoa appearing to be of less importance (see review by [18]). The biohydrogenation of unsaturated fatty acids involves several biochemical steps. Polan et al. [19] were among the first to propose that different populations of bacteria were necessary for the complete hydrogenation of polyunsaturated fatty acids. Kemp and Lander [20] divided the bacteria into two groups based on the reactions and end-products of biohydrogenation. Group A bacteria hydrogenate linoleic and linolenic acid only as far as *trans*-11 18:1. The second population, Group B bacteria, is capable of hydrogenating monounsaturated fatty acids and linoleic acid completely to stearic acid. Without group B bacteria, a complete hydrogenation of polyunsaturated fatty acids is not possible [20]. An alternative description of biohydrogenating bacteria based on their correct taxonomy has been proposed by Lourenço et al. [21].

**Fig. 1:**
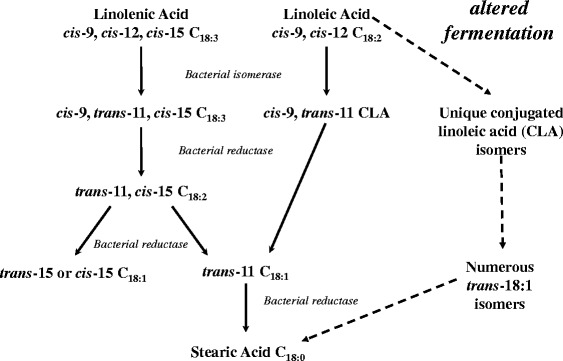
Steps in the biohydrogenation of linoleic and linolenic acid by rumen microorganisms. Various proportions of each intermediate are produced depending on rumen conditions. Adapted from [1, 27, 4]

The sequence of the biohydrogenation of linoleic acid and linolenic acid is presented in Fig. 1. The initial biohydrogenation step of fatty acids containing a *cis-*9, *cis-*12 double bond system is the isomerization of the *cis*-12 double bond by linoleate isomerase (EC 5.2.1.5). The isomerase has been partially purified and kinetic properties characterized in a limited number of bacterial species [14, 20, 22, 23]. In the biohydrogenation of linoleic acid, the second reaction is a reduction resulting in conversion of CLA to *trans*-11 18:1 followed by hydrogenation to stearic acid (Fig. 1). Similar to linoleic acid, biohydrogenation of linolenic acid begins with an isomerization followed by a sequence of hydrogenations, and terminates with formation of stearic acid. As biohydrogenation reduces unsaturated fatty acid availability for absorption, feeding dairy cows with polyunsaturated fatty acid-rich feeds, including fresh grasses, does not easily increase the quantities of these fatty acids in milk fat of dairy cows.

Supplementing the diet of dairy cows with oils and full-fat oilseeds containing unsaturated fatty acids can increase milk fat concentrations of polyunsaturated fatty acids [1], but this is not without risks. Supplementing polyunsaturated fatty acids to the diet increases the potential for a shift in biohydrogenation pathways that results in the production of biohydrogenation intermediates of linoleic acid that are known to cause milk fat depression (Fig. 1) and pathways of linolenic acid biohydrogenation are also likely to be affected. A variety of diets are known to cause changes in milk fat composition and low milk fat yields, but are typically categorized as diets high in readily digestible carbohydrates and low in effective fiber (high grain/low forage) and supplemented with polyunsaturated fatty acids [24, 25]. These diets modify the rumen environment initiating altered pathways of biohydrogenation and increased rumen outflow of *trans*-isomers. The level of milk fat reduction can be greater than 50 % without changes in milk protein or lactose concentrations [26].

The biohydrogenation theory of milk fat depression proposes that the production of *trans*-intermediates of altered rumen biohydrogenation pathways negatively impact synthesis of milk fat in the mammary gland [26]. Conjugated linoleic acids are octadecadienoic acids with conjugated double bonds and are primarily found in ruminant fats as a result of linoleic acid biohydrogenation [27]. While conjugated linoleic acids are purported to have numerous biological effects, the *trans*-10, *cis*-12 isomer is a potent inhibitor of milk fat synthesis [28]. Peterson et al. [29] demonstrated a curvilinear relationship between the dosage and milk fat yield of *trans-*10, *cis-*12 conjugated linoleic acid and the reduction in milk fat yield. The relationship exists between *trans*-10, *cis*-12 conjugated linoleic acid and milk fat yield in diet-induced milk fat depression as well. Lock et al. [30] examined *trans*-10 18:1, a downstream intermediate of *trans*-10, *cis*-12 conjugated linoleic acid biohydrogenation, for its potential as an additional fatty acid inhibiting milk fat synthesis. In this experiment, 42.6 g/d *trans*-10 18:1 was compared with 4.3 g/d *trans*-10, *cis*-12 conjugated linoleic acid, and fatty acids were infused for 4 days. No reduction in milk fat was observed with the *trans*-10 18:1 treatment. Authors concluded that *trans*-10 18:1 was a marker of altered biohydrogenation and did not appear to have a role in inhibition milk fat synthesis [30].

The presence of docosahexaenoic acid (*cis*-4*, cis*-7, *cis*-10, *cis*-13, *cis*-16, *cis*-19 22:6) in polyunsaturated fatty acids supplements can influence rumen biohydrogenation of dietary fatty acids. Klein and Jenkins [31] demonstrated that docosahexaenoic acid itself is not converted to *trans*-11 18:1, but modifies biohydrogenation of other long-chain polyunsaturated fatty acids present in the rumen, such as linoleic and linolenic acids. Docosahexaenoic acid promotes accumulation of vaccenic acid (*trans*-11 18:1) during linoleic acid biohydrogenation, but the presence of other fatty acids in polyunsaturated fatty acids supplements interact with dietary linoleic acid to increase *trans*-10, *cis*-12 conjugated linoleic acid production [32–34]. Rumen protection can circumvent interacting factors that influence biohydrogenation and prevent milk fat depression.

### Rumen protection of polyunsaturated fatty acids from biohydrogenation

Increasing the post-ruminal absorption of polyunsaturated fatty acids might have implications for improving dairy cow reproduction as described by Santos et al. [35] in their review of the effects and benefits of long chain fatty acids on reproduction. For cows, n-3 fatty acid supplements have been purported to improve reproductive efficiency of dairy cattle. N-3 polyunsaturated fatty acids inhibit prostaglandin-F_2α_ synthesis through competition with arachadonic acid as a precursor for prostaglandin synthesis [36–38]. In some studies, feeding n-3 fatty acids to cows reduced prostaglandin secretion and improved fertility and embryo survival [35].

Rumen protection increases polyunsaturated fatty acid flow to the duodenum and absorption by the intestines without impacting fermentation [39]. Technologies promising protection from biohydrogenation include coating polyunsaturated fatty acids with formaldehyde-treated casein, lipid encapsulation, or structural change using calcium salts and fatty acids amides to resist microbial enzymes [39]. Whole oilseeds and calcium salts of fatty acids provide consistent duodenal flows, but do not provide a protected source of n-3 polyunsaturated fatty acids [40]. Lipid encapsulation is a method that may allow n-3 polyunsaturated fatty acids to remain protected in the rumen, avoid biohydrogenation, and be available for absorption and utilization [41, 42]. Rumen-protected fat sources that deliver consistent duodenal flows of n-3 polyunsaturated fatty acids remain to be elucidated and commercially developed.

### Lipid absorption and secretion by the intestine

The absorption of long-chain fatty acids has been extensively reviewed [43–45]. Long-chain fatty acids reach the intestine largely as saturated free fatty acids affixed to the surface of feed particles and bacteria, or as components of microbial phospholipids and sterol esters [8]. Protected fatty acids delivered to the small intestine as intact triglycerides are digested by pancreatic lipase and colipase as they are in simple stomached animals, releasing free fatty acids and monoacylglycerols [44]. Bile salts secreted in the duodenum separate fatty acids from particles by their detergent action, forming micelles via lysolecithin produced by phospholipase A_2_. These components are absorbed by diffusion when the micelle moves across the unstirred water layer of the intestinal epithelium [8]. Fatty acid digestibility may decline with increased fatty acid intake as micelle formation becomes limiting [44].

In the enterocyte, long-chain fatty acids are esterified to form cholesterol esters, phospholipids, and triglycerides. Micelles form containing a triglyceride and cholesterol ester core with a surface monolayer of phospholipids and unesterified cholesterol. Apolipoproteins A and B (characteristically Apo B48; [44]), made in the intestine, are complexed with these lipids into very low-density lipoproteins and secreted into lymph, entering the bloodstream at the thoracic duct [46–48]. Other apolipoproteins are synthesized in the liver and transferred from high density lipoproteins in circulation. Apo CI to CIV are co-factors for lipoprotein lipase and Apo E is required for liver uptake; allowing lipoproteins to control lipid utilization in energy metabolism [44]. Chylomicrons are the largest and least dense (<0.95 g/mL) of the lipoproteins and are secreted by the enterocyte following a meal; however, very low-density lipoprotein (0.95–1.006 g/mL) secretion is predominant in ruminants due to the low fat content of their diets [44, 49].

Though triglycerides are the major lipid class found in lymph of simple stomached animals, the proportion of phospholipids to triglycerides is higher in ruminants [44]. Increased dietary fat or rumen outflow of polyunsaturated fatty acids increases chylomicron secretion, but the triglyceride proportion remains unchanged, meaning the surface monolayer components (phospholipids and unesterified cholesterol) are increased during fatty acid re-esterification in the enterocyte [43]. Bolus feeding of lipids is known to increase the triglyceride portion of very low-density lipoproteins [44]. However, bolus feeding of lipids disturbs rumen function by altering biohydrogenation and reduces DMI. Litherland et al. [50] demonstrated that reductions in DMI may be more pronounced with unsaturated free fatty acids rather than unsaturated triglycerides reaching the duodenum as a result of feeding increased quantities of supplemental fat.

### Systemic lipid transport

Unlike simple stomached animals, synthesis and secretion of very low-density lipoproteins by the bovine liver is minimal [44, 51]. This is related to the continuous flow of digesta in ruminants as opposed to the boluses associated with meals consumed by nonruminants. As triglyceride proportion diminishes, lipoprotein density increases and they contain greater concentrations of cholesterol esters, phospholipids and apolipoproteins. Intermediate-density lipoproteins and low-density lipoproteins are remnants of extensive very low-density lipoprotein triglyceride hydrolysis, but both classes have minimal concentration in the plasma of lactating cows [44]. High density lipoproteins are the major plasma lipoprotein in ruminants and are secreted by the liver as a reverse cholesterol transport system, returning excess peripheral cholesterol to the liver for bile secretion [44]. Rapid metabolism of very low-density lipoproteins (within 5 min) during lactation is due to triglyceride hydrolysis, but the metabolic half-life of low-density lipoproteins and high-density lipoproteins is much greater [49]. In fact, the fractional removal rate of very low-density lipoproteins is approximately 10-fold greater than that of low-density lipoproteins [47]. Supporting this rapid turnover, the triglyceride component of very low-density lipoproteins is a precursor for milk fat synthesis in addition to plasma free fatty acids. Harvatine and Bauman [52] demonstrated a slight lag in conjugated linoleic acid enrichment of and clearance from plasma phospholipids compared to triglycerides with abomasal infusion. This lag in phospholipid clearance supports the decreased turnover rate for high-density lipoproteins compared to very low-density lipoproteins in plasma.

Although feeding supplemental polyunsaturated fatty acids can overwhelm the normal esterification capacity of the small intestine allowing increased incorporation of polyunsaturated fatty acids into triglycerides secreted from enterocytes [44], partitioning of polyunsaturated fatty acids into plasma lipid fractions that are less available to the mammary gland is more common [4]. Previous results have demonstrated that supplemental, very long-chain, n-3 fatty acids are primarily transported in the phospholipids or cholesterol ester fraction of blood, making them largely unavailable to the mammary gland for enrichment of milk fat [53–55]. Christie et al. [56] hypothesized selective hydrolysis of very low-density lipoproteins triglycerides at the liver may protect polyunsaturated fatty acids from nonessential functions. Mammary uptake of triglyceride fatty acids from plasma is dependent upon the action of mammary lipoprotein lipase on chylomicrons and very low-density lipoproteins [46, 57]. The low transfer of polyunsaturated fatty acids to milk might be explained by the incorporation of polyunsaturated fatty acids into phospholipids and cholesterol esters at the small intestine or liver limiting their uptake by the mammary gland [58], although some data suggest that phospholipid fatty acids can contribute to the milk fatty acids [59–61].

Additionally, clearance of polyunsaturated fatty acids from triglycerides transported in very low-density lipoproteins may not occur via lipoprotein lipase. In studies utilizing triglyceride emulsions designed to model very low-density lipoproteins, the rate of clearance is fastest for fish oil emulsion particles, followed by medium-chain triglycerides and last, long-chain triglycerides emulsion particles [62]. Multiple pathways including lipoprotein lipase, apolipoprotein E, low-density lipoprotein receptor, and the lactoferrin-sensitive pathway control removal of long-chain triglycerides from emulsions containing primarily n-6 fatty acids [63, 64]. Park et al. [65] proposed increased margination of n-3-rich particles reflected activation of lipoprotein lipase. Margination is particle attachment to endothelium-bound lipoprotein lipase during lipolysis. Qi et al. [63] then suggested lipoprotein lipase primarily functions as a “bridge” protein to mediate fatty acids uptake through membrane-anchoring. Removal of n-3 particles was less affected by this mechanism and independent of apolipoprotein E, low-density lipoproteins receptor, and lactoferrin. In fact, Murray-Taylor et al. [64] demonstrated that the uptake of n-3 emulsion particles depends upon cell surface proteoglycans and non-low-density lipoprotein receptor cell surface anchoring. These studies might suggest mammary uptake mechanisms present in the lactating dairy cow may conserve essential fatty acids by limiting uptake of n-3 polyunsaturated fatty acids.

### Intracellular fatty acid transport

Following hydrolysis of triglycerides by mammary lipoprotein lipase, locally generated free fatty acids must cross the plasma membrane to enter the cell. There is considerable debate as to whether long-chain fatty acid transfer, especially in the case of polyunsaturated fatty acids, requires transport proteins or occurs through diffusion through the lipid bilayer. In nonruminants, rapidly facilitated protein-mediated uptake occurs through the fatty acid translocase/CD36 membrane receptor and fatty acid transport proteins, and CD36 is found in the milk fat globule membrane [66]. In adipocytes, the fatty acid translocase/CD36 complex is located within cavaeolae, flask-shaped lipid rafts rich in sphingolipids and cholesterol that create unique membrane domains [67]. The polyunsaturation of docosahexaenoic acid and its opposing interaction with sphingolipids and cholesterol cause it to disrupt lipid rafts [68]. It is possible that disruption of the size and distribution of lipid rafts by docosahexaenoic acid alters the ability of lipoprotein lipase to anchor very low-density lipoproteins for uptake of fatty acids by fatty acid translocase/CD36 or that the complex itself is disrupted. This might explain why transfer efficiencies for other n-3 polyunsaturated fatty acids, such as linolenic acid, resemble linoleic acid, another 18 carbon fatty acid.

As these long-chain fatty acids enter the cell, long-chain acyl-CoA synthetases (**ACSL**) catalyze the synthesis of fatty acyl-CoA, the substrates for both catabolic and anabolic pathways in the cell. The predominant isoform in bovine mammary tissue is ACSL1, and it may have a role in milk fat synthesis [66]. These ACSL isoforms may have overlapping substrate specificity, but it is possible that tissue specific distribution of each isoform allows for regulation of fatty acid fate within the cell.

Intracellular fatty acid binding proteins (**FABP**) are cytoplasmic proteins that are hypothesized to be essential for fatty acid transport and metabolism within the cell by accelerating long-chain fatty acid uptake and targeting fatty acids to intracellular organelles. Fatty acid binding proteins are over 25 times more effective than albumin in increasing long-chain fatty acid solubility in the cytoplasm and may target transfer of long-chain fatty acids to acceptor membranes through direct membrane interaction [69]. Intracellular fatty acid trafficking is quite complex, and functional redundancy and cooperation exists among several proteins, including others such as ACSL and fatty acid transport proteins which also determine metabolic fates of long-chain fatty acids [70]. There are also distinct differences in the function and binding activity of the nine FABP isoforms identified [71].

A 15 kD protein, FABP3 is highly expressed in cardiac and skeletal muscle, and has been linked to fatty acid trafficking, metabolism, and signaling. It was originally isolated in the mammary gland as the primary component of mammary-derived growth inhibitor as reviewed by Mather et al. [72]. Characterization of mammary-derived growth inhibitor revealed it to be a complex of FABP3 with some FABP4 [73–75]. The mammary-derived growth inhibitor complex inhibits cell proliferation in mammary epithelial cell lines *in vitro* and may play a role in the onset of differentiation [75, 76]. These effects of FABP3 may be independent of its fatty acid binding effects. Clark et al. [77] determined FABP3 lacks an N-terminal signal peptide, and it is not known if the protein complex is secreted *in vivo*.

According to Bionaz and Loor [66, 78], temporal patterns of gene expression reveal that FABP3 and FABP4 are highly expressed in bovine mammary tissue during early lactation, which follows the temporal pattern of MDGI expression in lactation observed by Politis et al. [76]. Bionaz and Loor [78] proposed a role of FABP3 in channeling long-chain fatty acids to stearoyl-CoA desaturase or triacylglycerol synthesis or in long-chain fatty acids activation of gene expression through peroxisome proliferator activated receptor-γ. Deletion of the FABP3 gene in mice causes defective fatty acid oxidation compensated by increased glucose utilization in heart and skeletal muscle [71]. While Clark et al. [77] reported no overt effects of gene deletion on mammary gland phenotype, FABP3 null mice appeared to have a lower percentage of total unsaturated fatty acids in milk fat. Overexpression bovine FABP3 in the non-secretory bovine mammary epithelial cell line, MAC-T, did not influence cellular fatty acid composition (Stamey and Corl, unpublished results). The human breast cancer cell line MCF7 does not express FABP3 and overexpression of FABP3 in MCF7 cells increased uptake of radioactively labeled palmitate and oleate, but other changes in fatty acid metabolism were not observed [79]. Kadegowda et al. [80] reported FABP3 expression increased in MAC-T cells when treated with rosiglitazone to activate proliferator activated receptor-γ and palmitate or stearate. Together, these findings support a potential role for FABP3 and FABP4 in lipid synthesis in the bovine mammary gland though the exact biological function remains to be elucidated.

### Milk fat synthesis

Both preformed fatty acids from plasma and *de novo* synthesized fatty acids in the mammary epithelial cell contribute to milk fat [57]. Polyunsaturated fatty acids in milk fat are derived from the preformed fatty acids in plasma. Preformed fatty acids arise from free fatty acids mobilized from adipose tissue or from dietary fatty acids transported in the triglyceride portion of very low-density lipoproteins. Acetate serves as the primary substrate for *de novo* lipogenesis and is converted to malonyl-CoA by acetyl-CoA carboxylase and is chain-elongated by fatty acid synthase catalyzing the addition of volatile fatty acids [81]. In adipose tissues, fatty acids are elongated to 16 carbons, forming palmitate, but in the mammary gland, short-chain fatty acids are rapidly released for esterification into triglycerides prior to complete elongation by chain-terminating transacylation by fatty acid synthase [82]. Saturated, long-chain fatty acids are shuttled to stearoyl-CoA desaturase, which inserts a double bond at the ninth position from the carboxyl end [57]. Other, non-saturated substrates can also be used by stearoyl-CoA desaturase including several *trans* 18:1 isomers [83]. Two *trans* 18:1 isomers, *trans*-11 and *trans*-7 18:1, produce conjugated linoleic acid isomers when desaturated at the ninth carbon [84, 83].

Preformed, *de novo*, and desaturated fatty acids are all delivered to the endoplasmic reticulum where they are esterified into triglycerides via the glycerol-3-phosphate pathway. As reviewed by Coleman and Mashek [85], glycerol-3-phosphate is converted to lysophosphatidic acid by glycerol-3-phosphate acyltransferase, adding a fatty acyl-CoA at the *sn*-1 position. Lysophosphatidic acid is converted to phosphatidic acid via acylation of the *sn*-2 position by acylglycerophosphate acyltransferase. Phosphatidic acid is a key intermediate in lipid synthesis as it is a branch point for phospholipid and triacylglycerol synthesis [85]. Multiple isoforms of acylglycerophosphate acyltransferase exist, and it is likely each isoform has specificity for incorporation of specific fatty acids in the *sn*-2 position. With the addition of another fatty acyl-CoA, phosphatidic acid is converted to diacylglycerol by phosphatidic acid phosphohydrolase.

To produce a triglyceride, the final fatty acyl-CoA, usually a short-chain fatty acid [86], is added by diacylglycerol acyltransferase. A single nucleotide polymorphism in diacylglycerol acyltransferase 1 in the mammary gland allows for genetic selection of cattle with enhanced milk fat yield [87]. Triglycerides accumulate within lipid droplets in the cell, which may contain portions of the rough ER, and gradually migrate to the apical plasma membrane of the mammary epithelial cell where they bud off with the plasma membrane forming the milk fat globule membrane [88].

### Transgenic approach to increasing milk fat polyunsaturated fatty acids

The limitations so far presented for transfer of polyunsaturated fatty acids, especially those of the n-3 series, into milk fat and the era of transgenics offer new approaches to alter milk fatty acid composition. The location of double bonds within the carbon backbone of polyunsaturated fatty acids makes them essential in the diet because mammals lack desaturase enzymes capable of adding double bonds beyond the ninth carbon. The only member of the animal kingdom known to include the delta-15 desaturase gene is the nematode, *Caenorhabditis elegans*. In recent years, efforts to incorporate this gene into the genome of mammals with expression specific to the mammary gland has been used to increase n-3 fatty acids in milk fat of mice [89]. Transgene expression was largely isolated to the mammary gland through the use of the β-casein gene promoter, although some expression was found in muscle also. Milk fat was separated into phospholipid and triacylglycerol fractions for fatty acid analysis and the greatest changes in polyunsaturated fatty acids were found in the phospholipid fraction. Significant increases linolenic and eicosapentaenoic were observed in both phospholipid and triacylglycerol fractions. Concomitant reductions in linoleic and arachidonic acids were observed in the phospholipid fraction. In a separate report, increases in n-3 docosapentaenoic acid in the triacylglycerol fraction and docosahexaenoic acid in the phospholipid fraction were observed [90]. Phospholipids make up a small fraction of milk fat and the presence of n-3 enrichment in this milk fat fraction limits overall changes in milk fatty acid composition. The enhanced localization of n-3 polyunsaturated fatty acid enrichment to the phospholipid fraction might be explained by substrate preference of the *C. elegans* desaturase for acyl lipids [91]. Despite the potentially low total enrichment of milk fat with n-3 polyunsaturated fatty acids, pups consuming the milk of transgenic dams had significantly enriched n-3 polyunsaturated fatty acids in brain lipids [90, 92].

There is now a report of a transgenic cow expressing the *C. elegans* desaturase [93]. To date, only the results for one cow are published. The chicken β-actin promoter was included allowing expression of the desaturase gene outside the mammary gland. Comparison with a cloned cow not expressing the transgene revealed increases in n-3 fatty acid content of milk fat and reductions in the n-6 series, but authors did not report overall milk fatty acid composition [93] and so it is difficult to determine the overall content of n-3 and n-6 fatty acids in milk fat. As the fatty acid substrate for the desaturase gene is linoleic acid and all polyunsaturated fatty acids are low in milk fat, the enrichment of n-3 fatty acids is likely limited by the availability of the substrate.

## Conclusions

Increasing polyunsaturated fatty acids in the milk fat of lactating dairy cattle is very challenging. The cow has multiple mechanisms capable of thwarting efforts to enrich milk fat with polyunsaturated fatty acids. Research into the mechanisms of rumen biohydrogenation, fatty acid digestion and absorption by the small intestine, and uptake and trafficking by the mammary epithelial cells of the lactating dairy cow has shed light on the obstacles that erode the effectiveness of simple supplementation strategies. These challenges have created an active area of research in the last half-century, increasingly motivated by consumer demand for healthier food choices. The more recent efforts at transgenic manipulation to bypass the evolutionarily engrained mechanisms of the cow might have potential to increase the polyunsaturated fatty acid content of milk fat, but even they present challenges not the least of which is the largely untested consumer tolerance for food from transgenic animals.
